# Two-Dimensional Differential Gel Electrophoresis to Identify Protein Biomarkers in Amniotic Fluid of Edwards Syndrome (Trisomy 18) Pregnancies

**DOI:** 10.1371/journal.pone.0145908

**Published:** 2016-01-11

**Authors:** Te-Yao Hsu, Hao Lin, Hsuan-Ning Hung, Kuender D. Yang, Chia-Yu Ou, Ching-Chang Tsai, Hsin-Hsin Cheng, Su-Hai Chung, Bi-Hua Cheng, Yi-Hsun Wong, An Kuo Chou, Chang-Chun Hsiao

**Affiliations:** 1 Department of Obstetrics and Gynecology, Kaohsiung Chang Gung Memorial Hospital, Kaohsiung, Taiwan; 2 Department of Pediatrics, Chang Bing Show Chwan Memorial Hospital, Chang Hwa, Taiwan; 3 Department of Anesthesia, Kaohsiung Chang Gung Memorial Hospital, Kaohsiung, Taiwan; 4 Genomic Medicine Research Core Laboratory, Kaohsiung Chang Gung Memorial Hospital, Kaohsiung, Taiwan; 5 Chang Gung University College of Medicine, Kaohsiung, Taiwan; Universidad Nacional de la Plata, ARGENTINA

## Abstract

**Background:**

Edwards syndrome (ES) is a severe chromosomal abnormality with a prevalence of about 0.8 in 10,000 infants born alive. The aims of this study were to identify candidate proteins associated with ES pregnancies from amniotic fluid supernatant (AFS) using proteomics, and to explore the role of biological networks in the pathophysiology of ES.

**Methods:**

AFS from six second trimester pregnancies with ES fetuses and six normal cases were included in this study. Fluorescence-based two-dimensional difference gel electrophoresis (2D-DIGE) and matrix-assisted laser desorption/ionization time-of-flight mass spectrometry (MALDI-TOF/MS) were used for comparative proteomic analysis. The identified proteins were further validated by Western blotting and the role of biological networks was analyzed.

**Results:**

Twelve protein spots were differentially expressed by more than 1.5-fold in the AFS of the ES pregnancies. MALDI-TOF/MS identified one up-regulated protein: apolipoprotein A1 (ApoA1), and four under-regulated proteins: vitamin D binding protein (VDBP), alpha-1-antitrypsin (A1AT), insulin-like growth factor-binding protein 1 (IGFBP-1), and transthyretin (TTR). Western blot and densitometric analysis of ApoA1, A1AT, IGFBP-1, and TTR confirmed the alteration of these proteins in the amniotic fluid samples. Biological network analysis revealed that the proteins of the ES AFS were involved mainly in lipid and hormone metabolism, immune response, and cardiovascular disease.

**Conclusions:**

These five proteins may be involved in the pathogenesis of ES. Further studies are needed to explore.

## Introduction

Edwards syndrome (ES, Trisomy 18) and Down’s syndrome (Trisomy 21) are the two most prevalence autosomal trisomies encountered at birth. ES is a severe chromosomal abnormality with a prevalence of about 0.8 in 10,000 infants born alive [1]. The prognosis of the fetus after delivery is poor, with at most 8% survival to one year [2]. The syndrome pattern comprises a recognizable pattern of major and minor anomalies, an increased risk of neonatal and infant mortality, and significant psychomotor and cognitive disability. The region of long arm of chromosome 18 extending from q11.2 has been proposed as the critical region for trisomy 18 phenotype, but some studies hypothesizing the presence of two critical regions along the long arm of chromosome 18 have been reported [3]. AF is known to contain a large amount of proteins, and their expressions reflect the genotypic constitution and phenotype of the fetus and regulate fetal-maternal physiological interactions [4]. An intricate balance of proteins is required throughout pregnancy and in cases of fetal genetic abnormalities this balance may be disturbed [5]. One of the major ways to channel transcriptional changes to the phenotypic level requires changes at the proteome level. It is therefore of great importance to understand whether aneuploidy-induced gene expression changes at the mRNA level generally translate also at the protein level [6]. Proteomics-based technology is one of a technique for protein identification in complex cellular systems is employed in pregnancy research [7]. Application of this technique in amniotic fluid (AF) analysis may recognize the mechanisms of pregnancy-related disorders and fetal genetic abnormalities. Although two-dimensional gel electrophoresis (2DE) has long been used to study differential proteomics, its reproducibility has always been a concern. In recent years, methodological improvements have contributed to more robust 2DE workflows such as the use of immobilized isoelectric focusing (IEF) strips, fluorescence-based two-dimensional difference gel electrophoresis (2D-DIGE), and new software tools [8,9]. The major benefits of 2D-DIGE over 2DE are the high sensitivity and linearity of the fluorescent dyes used, its straight forward protocol, and its significant reduction of inter-gel variability, which increases the possibility of identifying biological variability and reduces bias from experimental variations. Furthermore, the use of a pooled-sample internal standard loaded together with the control and study samples increases quantification and statistical accuracy [10,11]. In the present study, we attempted to identify candidate proteins associated with ES pregnancies from amniotic fluid supernatant (AFS) using fluorescence-based 2D-DIGE.

## Methods and Materials

### Sample Collection and Preparation

AF samples (8–10 ml) were obtained by amniocentesis from women at 16–18 weeks of gestation undergoing prenatal diagnosis at Kaohsiung Chang Gung Memorial Hospital, mostly due to advanced maternal age or an increased risk of Down’s in maternal serum, after written informed consent had been obtained. This study was approved by the Ethics Committee of Chang Gung Memorial Hospital. Approval from the institutional review board was obtained for the analysis of this series. AF samples were collected by our cytogenetic laboratory and centrifuged (1200x, 10mins, centrifuge (KUBOTA 5220 Japan)) to collect amniocytes for cytogenetic analysis. Karyotype analysis was performed on at least 20 metaphase cells, using the Wright technique for Giemsa-banding.

The cell-free supernatants were stored at -80°C until use. Six samples came from pregnancies that, as shown by conventional cytogenetic analysis, carried fetuses with ES, and six samples came from pregnancies with chromosomally normal fetuses. The most commonly identified ultrasound abnormality with trisomy 18 was hydrops fetalis (4/6, 66.7%). Congenital cardiac abnormality (3/6, 50%), ventriculomegaly (2/6, 33.3%), and clubbing foot (2/6, 33.3%) were the next most frequently seen ultrasound findings, followed by cleft lip & palate (1/6, 16.7%) and acrania (1/6, 16.7%). Post-abortal phenotype included hydrops fetalis, clubbing foot, cleft lip & palate, and acrania. Amniotic fluid supernatants were precipitated using three volumes of 100% ice-cold acetone at -20°C overnight, and the pellets were suspended in 50μl DIGE lysis buffer consisting of 30mM Tris-HCl (pH8.5), 7M urea, 2M thiourea, 4% CHAPS (3-[(3-Cholamidopropyl) dimethylammonio]-1-propanesulfonatehydrate), 0.4% DTT (Dithiothreitol), 10 μl/mL of protease inhibitor cocktail and 10 μl/mL of phosphatase inhibitor cocktail (Thermo Scientific, Rockford, USA). After centrifugation at 14,000 × *g* for 10 minutes (RCF; relative centrifugal force), the supernatants were collected. All supernatants were depleted for albumin and IgG with an Albumin and IgG Depletion Kit (Sigma-Aldrich, Missouri, USA). Samples were then desalted using a 2-D Clean Up Kit (GE Healthcare Bio-Sciences, Piscataway, USA) and dissolved in DIGE lysis buffer according to the manufacturer’s instructions. Protein concentration was determined using a 2-D Quant Kit (GE Healthcare Bio-Sciences, Piscataway, USA).

### Fluorescence Two-Dimensional Differential In-Gel Electrophoresis (2D-DIGE)

#### Protein labeling & 2-DE

AF proteins (50 μg) were labeled with Cy Dye DIGE Fluor minimal dye (GE Healthcare Bio-Sciences, Piscataway, USA) at a concentration of 400 pmol of dye/50 μg of protein. Samples were labeled Cy3, Cy5, or Cy2 (reference pool), and all three labeled samples were multiplexed and resolved in one gel. Labeled proteins were desalted by 2-D Clean Up Kit and dissolved in DIGE lysis buffer. For each gel, total 150 μg of protein were pooled in 350 μl rehydration buffer and applied to an immobilized 18 cm pH 4–7 linear gradient IPG strips (GE Healthcare Bio-Sciences, Uppsala, SWEDEN). The dried IPG strips were rehydrated with a 350 μl labeled sample aliquot, covered with Plus One Dry Strip Cover Fluid (GE Healthcare Bio-Sciences, Uppsala, SWEDEN) and incubated for 12 h at 20°C in the dark. The IPG strips were focused on an EttanIPGphor 3 (GE Healthcare Bio-Sciences) using a stepwise gradient of increasing voltage for a total of approximately 50000 voltage.

On completion of the first-dimension IEF, the IPG strips were equilibrated first in equilibration buffer containing 100 mM DTT for 15 min, followed by equilibration buffer containing 110 mMIAA (Iodoacetamide) for a further 15 min. The second dimension protein separation was performed on 12.5% DIGE Gels and DIGE buffer Kit (GE Healthcare Bio-Sciences, Uppsala, SWEDEN) using an EttanDALTsix electrophoresis system (GE Healthcare Bio-Sciences). The IPG strips were embedded on top of the gels with 0.5% w/v agarose, and the electrophoresis was conducted at 1 W/gel for 15–17 hours. The gels were run at 15°C.

#### Gel scanning and DIGE analysis

Images of the combined Cy2, Cy3 and Cy5 labeled proteins in each gel were scanned in a Typhoon 9400 scanner (GE Healthcare Bio-Sciences) using appropriate lasers and filters with a PMT (Photomultiplier Tube) voltage between 480 and 600 V. Images were prepared for further analysis using ImageQuant TL software prior to final analysis by Decyder™ software (GE Healthcare) using both the Differential In-gel Analysis (DIA) and Biological Variation Analysis (BVA) modules. Spots with a fold change of more than ±1.5 were considered to be differentially expressed, and subjected to in-gel digestion after silver staining as described below.

### Preparation of proteins for MALDI-TOF TOF/TOF analysis

#### Silver stain

At the end of scanning, proteins were fixed with 50% ethanol containing 10% acetic acid, and the gels were stained in a freshly made ammoniacal silver nitrate solution. The silver-stained gel images were scanned using an ImageScanner (GE Healthcare Bio-Sciences).

#### In-gel digestion

The spots were excised from the gels using a pipet tip with large orifice (1026-290-020, Labcon, San Rafael, USA), sliced into a 0.6ml micro centrifuge tube (Axygen, California, USA) and then washed three times in MilliQ water. The gel pieces were then destained with 30mM potassium ferricyanide (Sigma-Aldrich, Missouri, USA) and 100mM sodium thiosulfate (Sigma-Aldrich, Missouri, USA), and washed three times with 200μl of a solution containing 50mM ammonium bicarbonate/100% acetonitrile (3:2). The gel pieces were then shrunk in 100% acetonitrile (Merck, Darmstadt, Germany) followed by drying under vacuum in an Eppendorf Concentrator 5301 (Eppendorf AG, Hamburg, Germany) at 30°C. The desiccated gel pieces were rehydrated in 3μl of freshly prepared enzyme solution (20 ng/μl of sequencing grade modified trypsin (Promega, Madison, WI) in 25 mM ammonium bicarbonate to cover the gel pieces) and incubated at 4°C for 1h. The gel pieces were then left overnight at 37°C after the addition of 3μl of 25 mM ammonium bicarbonate to keep the gel wet. After 16 h, 2μl of 100% acetonitrile with 1% trifluoroacetic acid (Sigma-Aldrich, Missouri, USA) was added to make a total volume of approximately 3μl. The mixture was then sonified for 10 mins and the supernatant recovered. The acetonitrile/acid step was repeated and the supernatant was pooled. The peptide mixtures were prepared for MALDI-TOF MS-MS/MS analysis.

#### MALDI-TOF TOF/TOF analysis

Samples were prepared for MALDI-TOF MS-MS/MS analysis using a freshly prepared matrix solution containing 1.5 mg/mL of α-cyano-4-hydroxycinnamic acid (CHCA) (Bruker Daltonics, Bremen, Germany), 0.1% TFA (trifluoroacetic acid), 80% CAN (acetonitrile), and 20% MilliQ water. Peptide calibration standard II mixture (Bruker Daltonics, Bremen, Germany) allows for calibration (5×250 calibration points) and testing of MALDI-TOF mass spectrometers at a mass range between ~700 and 4000 Da. The standard calibration mixture contains nine standard peptides: Bradykinin 1–7, Angiotensin II, Angiotensin I, Substance P, Bombesin, Renin Substrate, ACTH clip 1–17, ACTH clip 18–39, and Somatostatin 28. A ten-fold dilution of the standard calibration mixture and peptide mixture was spotted onto a freshly cleaned anchor chip 600/384 sample plate and allowed to crystallize, and was then used as the matrix solution onto a sample plate and dried at room temperature. Peptide mixtures were analyzed with a matrix-assisted laser desorption tandem TOF mass spectrometer equipped with a 50 Hz 332 nm N_2_ laser (Ultraflex II MALDI-TOF-TOF MS/MS) (Bruker Daltonics, Bremen, Germany). Peptide matches and protein searches were performed automatically using MASCOT software (Matrix Sciences, London, UK). For peptide identification, monoisotopic masses were used and a mass tolerance of 50 ppm was allowed. All extraneous peaks, such as trypsin autodigests, matrix, and keratin peaks were not considered for protein searches. Only one miscleavage was allowed. The peptide masses were compared with the theoretical peptide masses of all available proteins from *homo sapiens* using the Swiss-Prot and NCBInr databases. A probability score with a *p* value less than 0.05 as identified by the software was used as the criterion for affirmative protein identification.

### Western blot analysis

Equal amounts of protein from the amniotic fluid samples were separated by 10%, 12%, 15% SDS-PAGE (Sodium dodecyl sulfate polyacrylamide gel electrophoresis), under reducing conditions. After electrophoresis, the proteins were electrotransferred to a Hybond-P PVDF transfer membrane (GE Healthcare Bio-Sciences, Buckinghamshire, UK) and blots were blocked for 1 h at room temperature with 5% nonfat milk in TBST buffer (50mM Tris, 150mM Nacl, 0.05% Tween 20, Adjust pH with HCl to pH 7.6). Membranes were then incubated overnight at 4°C with the appropriate dilution of rabbit polyclonal antibodies to insulin like growth factor binding protein 1 (IGFBP-1) (Abcam, Cambridge, UK), rabbit monoclonal antibodies to transthyretin (Epitomics, California, USA), and mouse monoclonal antibodies to alpha 1 antitrypsin (Abcam, Cambridge, UK) in the blocking buffer. After TBST buffer washing, the membranes were incubated with goat anti-rabbit IgG-HRP (Santa Cruz Biotechnology, CA, USA) or goat anti-mouse IgG-HRP (Santa Cruz Biotechnology, CA, USA) as secondary antibodies. Bound antibodies were detected by ECL (Enhanced chemiluminescence) Plus Western blotting detection reagents (GE Healthcare Bio-Sciences, Buckinghamshire, UK). Equal protein loading was confirmed by exposure of the membranes to mouse monoclonal antibodies to human IgG (Santa Cruz Biotechnology, CA, USA). Western blots were scanned and images were quantified for protein content using ImageJ software. ImageJ software was designed at National Institute of Health as open tools for the analysis of scientific images [12]. Mean protein quantification after Western blot analysis was performed by three independent experiments.

### Statistical analysis

Means of protein quantification were compared for both the patient and control groups. The values were expressed as mean ± standard deviation (SD). The significance of difference in means was tested by the paired Student’s *t*-test. A *p*-value of less than 0.05 was considered significant. Statistical analyses were performed using SPSS analytical software (SPSS Inc., Chicago, IL, USA).

### Bioinformatics analysis

In order to identify relevant pathways in the pathogenesis of ES, we used MetaCore (GeneGo, St. Joseph, MI) to elucidate the biological processes of differentially expressed proteins in the AFS of ES fetuses. MetaCore is an integrated knowledge database and software suite for pathway analysis of experimental data, and is based on a proprietary manually curated database of human protein interactions and metabolism. This data analysis tool enables statistical tests and scoring for network relevance to the dataset, functional processes, cellular pathways and transcription factors. We uploaded the differently expressed proteins using their Swiss-Prot IDs to MetaCore for analysis. Biological process enrichment was analyzed based on GO Ontology processes. For network analysis, the shortest path algorithm was used to map the shortest path for interaction.

## Results

### Protein differential displays of AFS from ES and normal pregnant women

**Fig 1:**
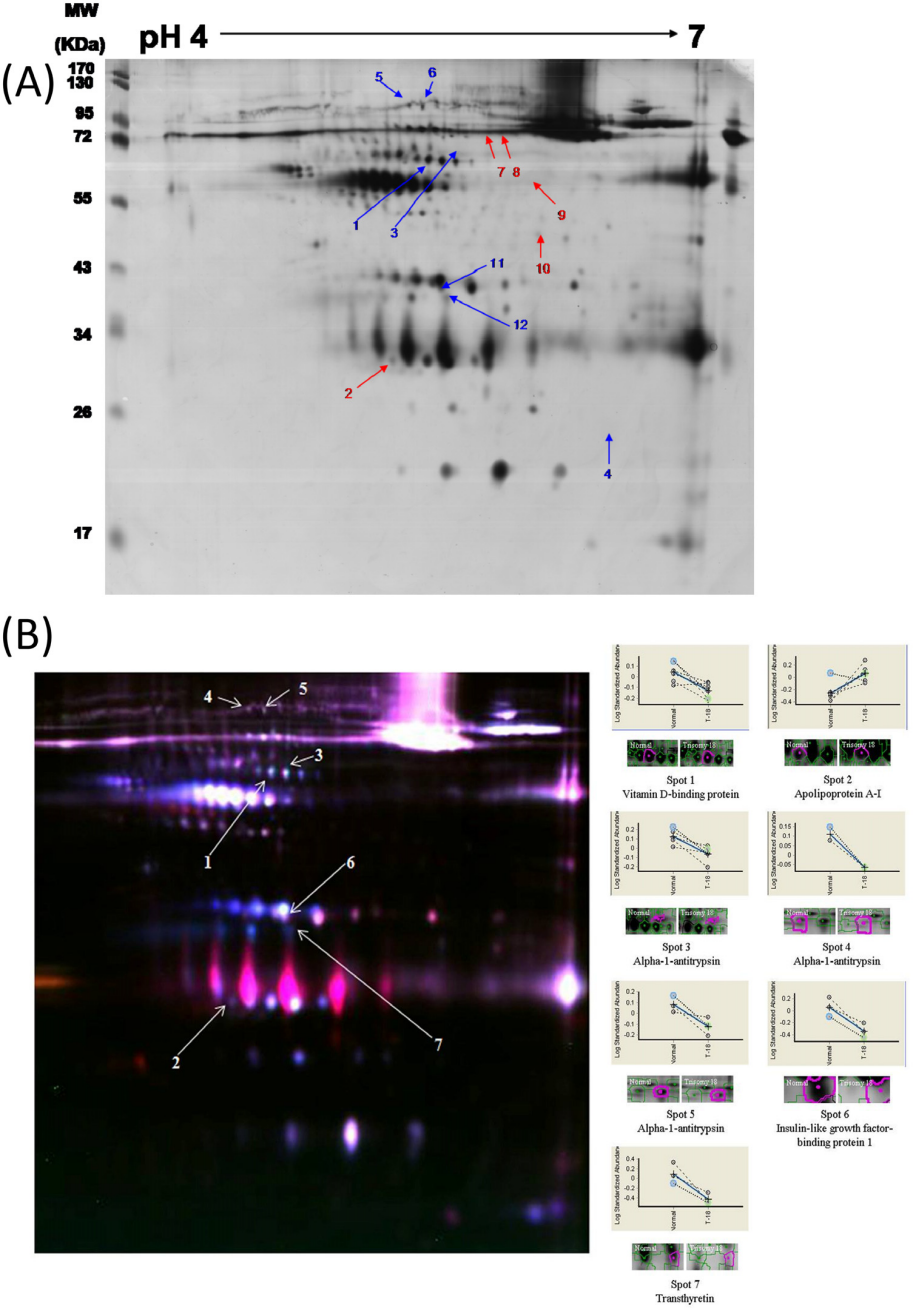
Protein differential displays of AFS from ES and normal pregnant. (A) 2D-DIGE gel stain by silver stain: Of these 12 protein spots, 5 were up-regulated and 7 were under-regulated in the ES subjects compared with the normal controls. Red arrow (spot 2, 7–9 and 10): up-regulated; Blue arrow (spot 1, 3–6 and 11–12):under-regulated. (B) 2D-DIGE images of AFS from women carrying ES and chromosomally normal fetuses: Spot 1: Vitamin D binding protein (VDBP); Spot 2: Apolipoprotein A1 (ApoA1); Spot 3–5: Alpha-1-antitrypsin(A1AT); Spot 6: Insulin-like growth factor-binding protein 1(IGFBP-1); Spot 7: Transthyretin (TTR).

Six samples came from pregnancies that, as shown by conventional cytogenetic analysis, carried fetuses with ES, and six samples came from pregnancies with chromosomally normal fetuses. 12 protein spots were differentially expressed by more than ±1.5-fold by Decyder™ software. Of these 12 protein spots, 5 were up-regulated and 7 were under-regulated in the ES subjects compared with the normal controls (Fig 1A).

### Protein identification

The 12 protein spots per gel were further excised for in-gel digestion by trypsin, and 7 of them were successfully identified by MALDI-TOF-MS. The 7 spots are markered on the combined 2D-DIGE image is shown in Fig 1B. The results of the database search showed that the 7 spots were matched to 5 unique proteins. One protein, apolipoprotein A1 (ApoA1), was found to be up-regulated, and the four other proteins, insulin-like growth factor-binding protein 1 (IGFBP-1), vitamin D binding protein (VDBP), transthyretin (TTR), and alpha-1-antitrypsin (A1AT) were under-regulated. According to their fold change, the one up-regulated (ApoA1) and three most significantly under-regulated proteins (A1AT, IGFBP-1, and TTR) were selected for Western blot verification. Table 1 summarizes the identified protein names, Swiss-Prot accession numbers, theoretical p*I*, molecular weight (Mw), MASCOT score (Ms), and protein coverage.

**Table 1:** Significant differentially expressed proteins identified in the amniotic fluid of women with Edwards syndrome fetuses.

Spot No.	SwissPort Entry Name	Protein Name	Theoretical p*I*/Mr	SequenceCoverage, MS%	MASCOT Score	Fold Change	T-test
**1**	**VTDB_HUMAN**	**Vitamin D-binding protein**	**5.40/52.9**	**50**	**108**	−**1.50**	**0.0052**
2	APOA1_HUMAN	Apolipoprotein A1	5.56/30.8	48	81	+2.03	0.0043
3	A1AT_HUMAN	Alpha-1-antitrypsin	5.37/46.7	59	153	−1.52	0.0090
4	A1AT_HUMAN	Alpha-1-antitrypsin	5.37/46.7	49	143	−1.50	0.039
5	A1AT_HUMAN	Alpha-1-antitrypsin	5.37/46.7	65	143	−1.59	0.038
6	IGFBP1_HUMAN	Insulin-like growth factor binding protein 1	5.11/27.9	38	83	−2.54	0.027
7	TTHY_HUMAN	Transthyretin	5.52/15.9	48	61	−3.52	0.022

MS = mass spectrometry; Mr = molecular weight.

### Confirmation of the identified proteins by Western blotting analysis

**Fig 2:**
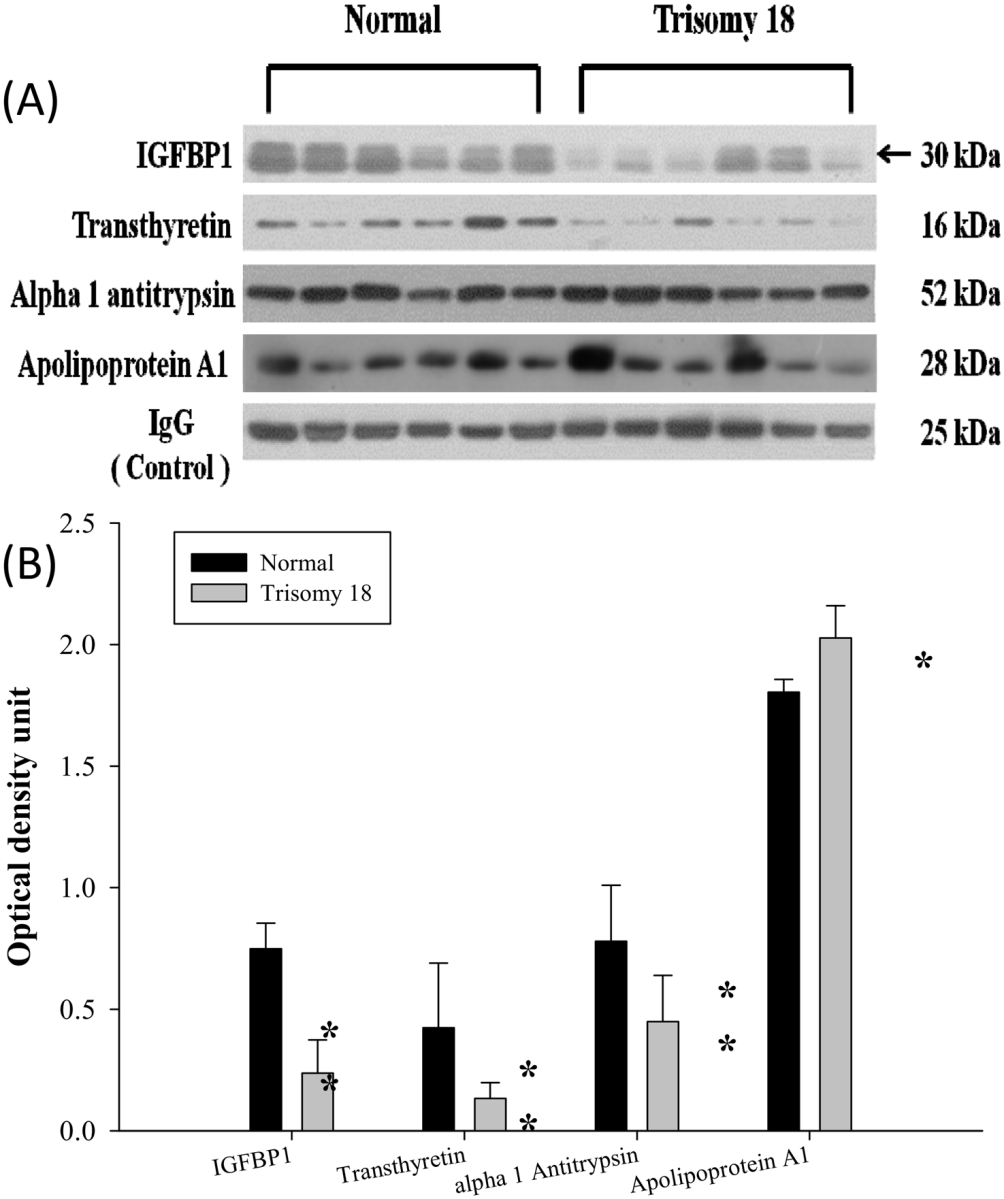
Western blot analysis of IGFBP-1(IGFBP-1 = IBP-1 = insulin growth factor binding protein 1, TTR, A1AT, and ApoA1in normal and ES AFS. (A) Equal protein amounts of AFS from the women carrying normal and ES fetuses were separated by gel electrophoresis and immunoblotted with the appropriate dilution of antibody. One membrane out of the four independent replicates is shown. The nonspecific bindings of human IgG was used as internal loading controls. (B) Quantification of protein content of IGFBP-1, TTR, A1AT, and ApoA1 using scanning densitometry. Each bar represents the mean (SD) of four independent experiments. Dark striped bars, normal; light bars, ES; **p <0.003, * *p* <0.02

Western blotting was used to test for ApoA1, A1AT, IGFBP-1, and TTR. The blots of each and the corresponding IgG are shown in Fig 2A. Quantification of protein expression by scanning densitometry showed that the ApoA1 protein was significantly increased, while the other three proteins (A1AT, IGFBP-1, and TTR) were significantly decreased in the amniotic fluid of ES pregnancies (Fig 2B).

### MetaCore analysis of biological network on a group of proteins

**Fig 3:**
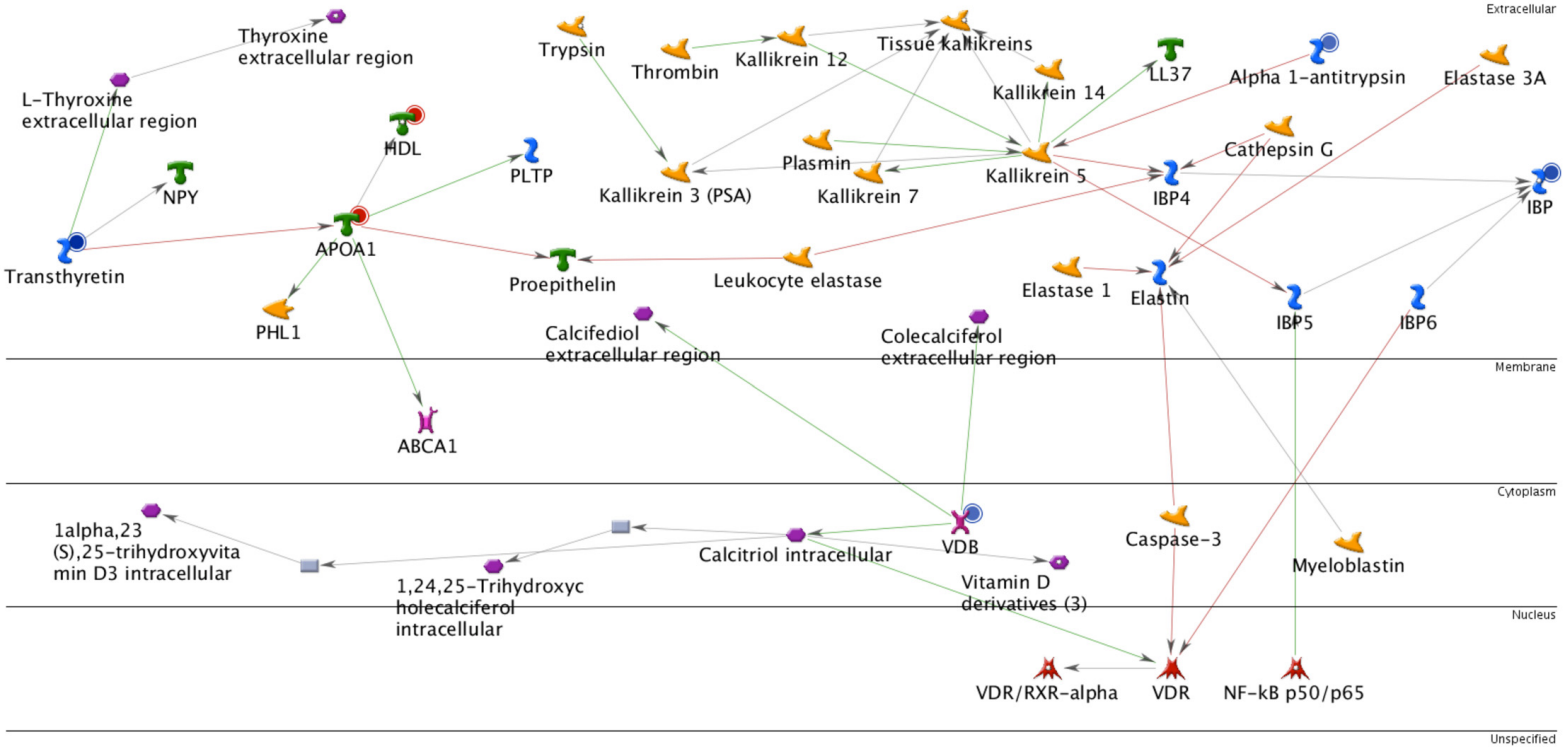
Biological network analysis of the differentially expressed proteins using MetaCore mapping tools. Five differentially expressed proteins in the pregnancies with Edwards syndrome (Trisomy 18) fetuses (as shown in red solid circles indicating over-expressed levels; blue solid circles indicating under-expressed levels) were found to interact with proteins in this network. Some proteins are shown as gene symbols: IBP = IGFBP-1 = insulin growth factor binding protein 1; APOA1 = apolipoprotein A1; VDB = vitamin D binding protein.

In the search for the biological network of the differentially expressed proteins using MetaCore mapping tools, we found that the five proteins identified (ApoA1, A1AT, IGFBP-1, VDBP, and TTR) may participate in the transcriptional activation of pathways in regulating cellular responses (Fig 3). Analysis of the functional network of these five proteins also lead to several processes involved in lipid and hormone metabolism, immune response, and cardiovascular diseases. The *p*-value for the resulting network was 4.79E-13, indicating that the probability of assembly from a random set of nodes (genes) was very low. The top 10 biological processes and diseases associated with the differentially expressed proteins in the pregnancies with ES fetuses are listed in Table 2.

**Table 2:** Top 10 biological processes and diseases that may be associated with the differentially expressed proteins in the women with Edwards syndrome (Trisomy 18) fetuses.

**Biologic processes**	***P* Value**
Response to estrogen stimulus	6.412E-08
Negative regulation of interleukin-1 beta secretion	1.672E-07
Cholesterol import	5.014E-07
Negative regulation of interleukin-1 secretion	5.014E-07
Sterol import	5.014E-07
Sterol transmembrane transport	5.014E-07
Negative regulation of very-low-density lipoprotein particle remodeling	1.003E-06
Hormone metabolic process	1.188E-06
Regeneration	1.355E-06
Negative regulation of cytokine secretion involved in immune response	1.671E-06
**Diseases**	***P* Value**
Liver Cirrhosis	5.355E-09
Cerebrovascular Disorders	5.599E-08
Cardiomyopathies	7.629E-08
Eye Diseases	2.254E-07
Stroke	3.533E-07
Aortic Valve Stenosis	4.198E-07
Heart Valve Diseases	8.463E-07
Rheumatic Diseases	2.932E-06
Tangier Disease	4.161E-06
Arthritis	4.501E-06

## Discussion

In this study, we demonstrated that differentially proteins are present in the amniotic fluid of ES pregnancies. One protein (ApoA1) was found to be up-regulated, and four proteins (IGFBP-1, VDBP, TTR, and A1AT) were found to be under-regulated by 2D-DIGE and MALDI-TOF/MS. Three of the under-regulated proteins (IGFBP-1, TTR, and A1AT) were further validated by Western blotting.

Proteomic analysis has been utilized in several studies to define diagnostic markers for aneuploid pregnancies. Among them, most have focused on DS pregnancies because it is the most common aneuploidy worldwide [12]. Only one previous study investigated protein expressions in AFS associated with ES. In that study, levels of apolipoprotein A1 (ApoA1), AP-3mu, and antitrypsin were significantly decreased in the AFS of fetuses with ES, whereas placental protein-14 was increased [13]. ApoA1 is a protein that helps to clear cholesterol from arteries [14]. It has also been reported to have anti-inflammatory properties and to help protect against Alzheimer’s disease [15]. According to its biological characteristics, it has been demonstrated that apolipoproteins play an important role in lipoprotein metabolism in the central nervous system. The finding of increased oxidation of ApoA1 is in line with recent reports highlighting that ApoA1 dysfunction may be linked to increased susceptibility to cognitive impairment [16]. ApoA1 has been found at decreased levels in subjects with a variety of neurodegenerative disorders including in the serum and cerebrospinal fluid of Alzheimer disease, Parkinson disease and DS with gout subjects. Perluigi M *et al* pointed out that AF ApoA1 increase oxidative damage is an early event in the DS pathogenesis and might contribute to the development of deleterious DS phenotypes [17]. The dysregulation of ApoA1 expression in AF may indicate a possible correlation between defective lipid metabolism or neurological deficits and ES in very early fetal development. It could also be related to ventriculomegaly. The evidence mentioned above indicating that ApoA1 may play a role in the pathogenesis of ES.

Other new findings in our study were the significant under-regulation of IGFBP-1, VDBP, TTR, and A1AT in the AFS of ES pregnancies. IGFBP-1 serves as a carrier protein for insulin-like growth factor 1 and 2 (IGF1 and IGF2), and binding of this protein prolongs the half-life of IGF1and IGF2 and alters their interaction with cell surface receptors in complex ways [18]. IGF1 and IGF2 are mitogenic polypeptides that have been shown to be important determinants of fetal growth during human pregnancy [19–20]. Animal experiments have shown that IGF-1and IGF-2 are principally involved in fetal growth and placental development, respectively [21,22,23]. Fetuses with ES are known to be small for gestational age in the first trimester [24], and therefore the expression of IGFBP-1 may be altered. Miell*et al* [25] was the first to find that maternal serum levels of IGFBP-1 in ES pregnancies were elevated compared to mothers with normal or DS fetuses. They hypothesized that un-elevated IGFBP-1 level in DS mother was due to the normal growth of fetuses during first trimester. Later, Tisi*et al* [26] also found an association between high second trimester level of AF IGFBP-1 and low birth weight in chromosomally normal fetuses. However, in a recent study by Sifakis*et al* [27] demonstrated that maternal serum levels of IGFBP-1 in ES, DS, and normal pregnancies at 11–13 weeks of gestation were not significantly different. In contrast, in our study, we found a decreased expression of IGFBP-1 in AFS of ES pregnancies. The large discrepancy among various studies may be explained by the complex ways that IGFBP-1 modulates IGF1 and IGF2 activities, involving both inhibiting and promoting actions [28].

Vitamin D-binding protein (VDBP) is a multi-functional plasma protein with many important functions. These include transport of vitamin D and its metabolites through the circulation, control of bone development, binding of fatty acids, sequestration of actin, and modulating immune and inflammatory responses [29]. This protein is encoded by the AFP (alpha fetoprotein) gene located on chromosome 4 [30]. Low circulating levels of maternal serum AFP are well known to be associated with pregnancies affected by DS and ES [31]. A previous study also showed that a decreased AFP expression may modify the distribution of other protein fractions in AF [32]. Whether or not the under-regulation of VDBP in the AF samples of ES pregnancies in our study contributed to the low level of AFP production requires further investigations. Another previous study also reported under-regulation of VDBP in AF samples in Klinefelter syndrome pregnancies [33]. However, the authors could not find any direct correlation between this protein and Klinefelter phenotypes.

Transthyretin (TTR), also known as prealbumin, is encoded by the TTR gene on 18q12.1. It serves as a serum and cerebrospinal fluid (CSF) carrier of thyroxine and retinol and is therefore important for normal fetal development. The liver secretes TTR into the blood, and the choroid plexus secretes TTR into the CSF [34]. TTR gene mutation is known to be associated with amyloid diseases [35], and low levels of TTR in the CSF have also been found to be associated with some neurological disorders such as schizophrenia [36]. Lolis*et al* [37] proposed that AF TTR expressed as a ratio of total protein may be a potential biomarker for certain fetal defects and complications of pregnancy but not specific to a particular disorder. Serum and AF TTR levels have been found to be up-regulated in DS pregnancies [12,38], however, expressions of TTR have not been reported in relation to ES pregnancies. To the best of our knowledge, the current study is the first to demonstrate an association of under-regulation of AF TTR with ES pregnancies.

Alpha 1-antitrypsin (A1AT) is an acute phase protein, the primary function of which is believed to be protection of tissues against the release of proteolytic enzymes following an inflammatory process [39]. As an acute phase protein, plasma A1AT concentrations can increase in response to stimuli such as infection or tissue trauma. Elevations of this protein in AF were also associated with severe preeclampsia in pregnant women in our previous study [40]. Decreased maternal serum A1AT levels have also been found to be correlated with fetal intrauterine growth retardation (IUGR) [41]. An association between maternal A1AT expression and fetal anomalies has also been reported. Increased A1AT expressions have been observed in AF samples obtained from pregnant women with Klinefelter syndrome and DS fetuses [32,37]. In our study, however, AF A1AT expression was decreased in ES pregnancies in agreement with the study by Wang *et al* [12]. The under-regulation of A1AT may be explained by IUGR of ES fetuses.

The functional network analysis of differentially expressed proteins in ES AFS using the MetaCore database showed that it might relate biological processes and diseases were lipid and hormone metabolism, immune responses, and cardiovascular diseases. Previous studies have demonstrated that cholesterol is an important lipid regulating steroid hormone synthesis, and its deficiency can cause several malformation syndromes including ES [42]. Other studies have also reported that T-lymphocyte, T-helper lymphocyte, and natural killer cell counts were significantly lower in ES fetuses than in gestational age-matched normal controls in the second trimester of pregnancy [43]. The majority of affected children die before the age of 1year because of the breakdown of the immune system leading to catastrophic infectious complications [44]. The results of the network analysis based on the differentially expressed proteins also related to several diseases that would be associated with ES, and most of our cases were related to hydrops fetalis and cardiovascular malformation, which is in agreement with the clinical characteristics of ES. Other related diseases such as liver cirrhosis have also been reported in association with ES [45].

Present study has several limitations that must be acknowledged. First, the case number to be investigated is too small. Second, validation of these molecules prospectively in different populations is lacking. Third, whether or not these proteins would cross the placenta barrier and be detected in maternal serum is not evaluated. Therefore, we do not know if these proteins could be utilized as serum biomarkers for early non-invasive prenatal diagnosis of ES.

In conclusion, the use of proteomic tools enables the identification of relevant protein markers that could be involved in the pathogenesis of ES fetuses. The combined use of proteomic tools and functional network analysis proved to be instrumental in the integral analysis of the differentially expressed proteins in this study for identifying the biological processes and relevant diseases that might be involved in ES. Although the discovery of these proteins holds much promise, further validation in prospective studies with a larger sample size are warranted.
